# Clove (*Syzygium aromaticum*) Pods: Revealing Their Antioxidant Potential via GC-MS Analysis and Computational Insights

**DOI:** 10.3390/ph18040504

**Published:** 2025-03-31

**Authors:** Abdelmuhsin Abdegadir Abdelmuhsin, Abdel Moniem Elhadi Sulieman, Zakaria Ahmed Salih, Meshari Al-Azmi, Naimah Asid Alanaizi, Ahmed Eisa Goniem, Mohammad Jahoor Alam

**Affiliations:** 1 Department of Biology, College of Science, University of Ha’il, Ha’il 81422, Saudi Arabia; abdelmuhsin@yahoo.com (A.A.A.); n.alenezy@uoh.edu.sa (N.A.A.); ahmedghoniem2010@gmail.com (A.E.G.); jahooralam@gmail.com (M.J.A.); 2 Department of Research and Training, Research and Training Station, King Faisal University, Alhsa 31982, Saudi Arabia; zakariasalih@yahoo.com; 3 Department of Information and Computer Science, College of Computer Science and Engineering, University of Ha’il, Ha’il 81422, Saudi Arabia; meshari.alazmi@kaust.edu.sa

**Keywords:** antioxidant, anti-inflammatory properties, shelf life, flavor, phenols

## Abstract

**Background:** *Syzygium aromaticum* is a tree whose aromatic dried flower buds are known as cloves. When it comes to phenolic chemicals, such as flavonoids, hydroxybenzoic acids, hydroxycinnamic acids, and hydroxyphenyl propane, clove is a major plant source of these substances. Finding out how effective clove buds are as antioxidants was the driving force behind this study’s GC-MS investigation and computational discoveries. **Methods:** This inquiry into clove pods focused on the chemical composition of clove using the GC-MS technique, as well as its antioxidant qualities and computational modeling. **Results:** This antioxidant may be more effective in lower doses than ascorbic acid (A.A.), butylate hydroxytoluene (BHT), and β-carotene, with 57.22 ± 0.41 mg QE/g of total phenols and flavonoids and 7.25 ± 0.12 mg GAE/g of clove extract. Phenols destroy free radicals, which boosts antioxidant activity. Flavonoids defend against ROS, which also boosts antioxidant activity. Clove pod GC-MS analysis identified 21 components, of which eugenol accounted for 58.86%. The absence of nitrogen and chlorine molecules emphasizes the composition’s organic nature. Eugenol, the major component of clove oil, is a phenolic molecule that binds strongly to bacterial enzymes such as DNA gyrase and dihydrofolate reductase. Docking experiments have shown that clove chemicals interact with acetylcholinesterase, a crucial enzyme in insect larvae, paralyzing and killing them. **Conclusions:** This study demonstrates the immense potential of plants in providing novel therapeutic and environmental solutions. We must support further research into nature’s inherent benefits. The extensive knowledge that can be gained from botany can be used to improve health, ecology, and sustainability.

## 1. Introduction

Historically, people have turned to medicinal plants for various medical needs. The remarkable in vitro efficacy of medicinal plants as antimicrobial agents, antiviral agents, antioxidants, and anticancer agents, as well as their myriad bioactive properties, has rekindled interest in these plants in the last few decades [1,2,3,4].

The highly esteemed spice clove, formally known as *S. aromaticum* (L.) (Merr. and L.M. Perry) has a long and celebrated history of use. The medicinal properties of cloves were known to ancient cultures. One of many spices used to flavor meals, it is also famous for alleviating pain, often targeting toothache. Still, it has many other benefits, the most known of which involve improving immunity and safeguarding from infections. It is believed that cloves’ anti-inflammatory and anti-free-radical properties may help alleviate various illnesses, supporting good lung function [5,6,7].

Cloves have several uses related to the respiratory system, including alleviating pain in the teeth and gums; warding off upper respiratory tract infections; and stopping the common cold, flu, cough, runny nose, and asthma attacks. Relieving toothaches is one of cloves’ most common uses. They have antibacterial and antiseptic properties, containing the anesthetic eugenol oil. They also help to prevent and treat stomach ulcers, improve sexual health, and aid people with diabetes in controlling their blood sugar levels, reducing the likelihood of complications [8,9,10].

They are a good source of vitamin C, which the body uses to fight free radicals. They also help with digestion, alleviate dental pain, and have a significant impact on fertility [11]. Cloves contain a major constituent compound called eugenol, which has been shown to act as a natural antioxidant [12,13]. Eugenol is clear or pale yellow, with a peppery taste and clove-like smell, and has been used to flavor food and drugs since the 19th century. It is used as a mild rubefacient in dentifrices and as an obtundent for hypersensitive dentine, caries, or pulp. The wide range of eugenol’s activities include its antimicrobial, anti-inflammatory, analgesic, and antioxidant effects. It has also been used in dental cement, analgesics, anesthetics, and zinc oxide-mixed temporary dental fillings [14].

Research on clove buds has been focused on their great antioxidant and antibacterial action, which has made them extensively employed in traditional medicine and food preservation. Rich in bioactive terpenoids and flavonoids, clove buds are the principal source of clove essential oil [15,16].

Cloves also have significant insecticidal properties. Eugenol inhibits acetylcholinesterase, an essential insect nervous system enzyme, causing paralysis and death in larvae. Caryophyllene interacts with detoxification enzymes, impairing larvae’s metabolization, and disrupts the juvenile hormone esterase, causing developmental delays and increased mortality. The combination of these compounds enhances their insecticidal properties, with a combination of eugenol and caryophyllene showing stronger inhibition [17,18,19]. This makes cloves a promising candidate as a natural preservative. Our research focused on identifying the proximate chemical composition, antioxidant capabilities, and GC-MS constituents of clove pods and computational modeling.

## 2. Results

### 2.1. Antioxidant Properties Results

The antioxidant activity of clove extract is shown in Table 1. We evaluated the scavenging activity of the total phenols, flavonoids, DPPH, β-carotene, and ABTS IC_50_ (mg/mL) compared with the existing reference molecules. The results suggest that low concentrations of this antioxidant may be more efficient than ascorbic acid (A.A.), butylate hydroxytoluene (BHT), and β-carotene. The clove extract had 7.25 ± 0.12 mg GAE/g and 57.22 ± 0.41 mg QE/g of total phenols and flavonoids, respectively. Phenols neutralize free radicals, increasing antioxidant activity. Flavonoids fight ROS and increase antioxidants. Table 1 also shows that the clove extract had a DPPH radical scavenging activity (IC_50_) of 0.08 ± 0.01 mg/mL, while BHT and ascorbic acid had IC_50_ of 0.024 ± 2 × 10^−4^ and 0.021 ± 5 × 10^−4^ mg. Lower IC_50_ values indicate higher antioxidant potential. Although clove extract has antioxidant capabilities, it was shown to be less efficient than BHT and ascorbic acid in this experiment, due to its higher IC_50_.

Clove extract, rich in phenolic components like eugenol, gallic acid, and flavonoids, is a potent antioxidant that neutralizes reactive oxygen species (ROS) to reduce oxidative stress. The DPPH assay was used to measure the antioxidants’ free radical scavenging activity, with eugenol being the main bioactive ingredient. Clove extract also effectively removed the ABTS•+ radical cation, a green–blue chromophore produced by potassium persulfate. Clove extract’s phenolic composition enhances its antioxidant activity, with higher inhibition values in the ABTS assay. This makes clove extract a potential food preservative and treatment for oxidative stress-related diseases, as it protects biological components from oxidative damage [20,21].

### 2.2. GC-MS of Clove Pods

**Figure 1:**
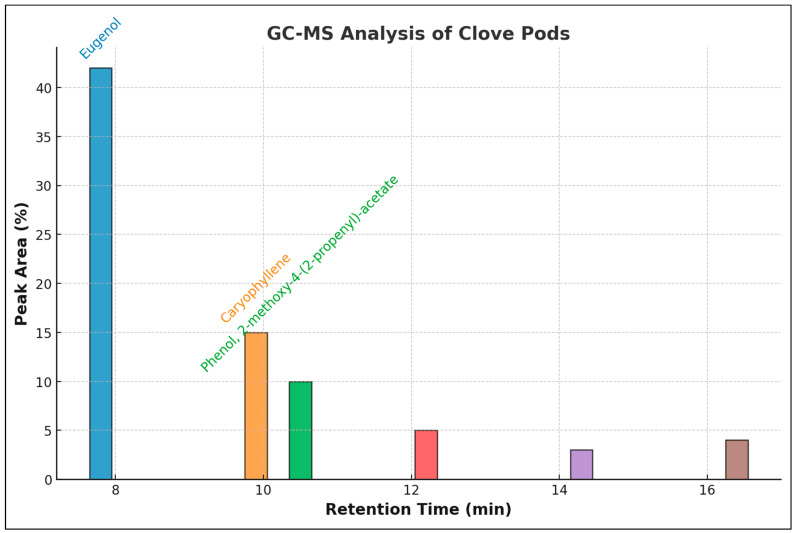
GC-MS chromatogram of clove (*S. aromaticum*) pods. GC-MS chromatogram of clove pod extract showing the major identified compounds. The x-axis represents retention time (minutes), while the y-axis shows the relative peak area (%). The labeled compounds correspond to their respective peaks, with color-coded text matching the bar colors for clarity.

Figure 1 and Table 2 show that 21 distinct chemicals were detected in clove pods using GC-MS analysis, with retention times ranging from 7.564 to 17.023 min. Eugenol accounted for 58.86% of the compounds, with caryophyllene constituting 14.72%, phenol 9.60%, humulene 3.62%, eugenyl acetate 3.13%, and n-hexadecanoic acid methyl ester 2.05%. The absence of chlorine and nitrogen compounds is notable. Three esters and three phenolic compounds, with concentrations ranging from 0.08% to 0.43%, were also detected in the clove pods during the examination.

The discovered phenolic chemicals ranged in concentration from 0.21% to 0.41% and included phenol, 4-(2-propenyl)-, 2-propenal, 3-phenyl-, and phenol, 2-methoxy-4-(2-propenyl)-acetate. No acetic acid, phenylmethyl ester, docosanoic acid, or n-hexadecanoic acid were discovered as esters.

### 2.3. Molecular Docking Analysis Results

**Figure 2:**
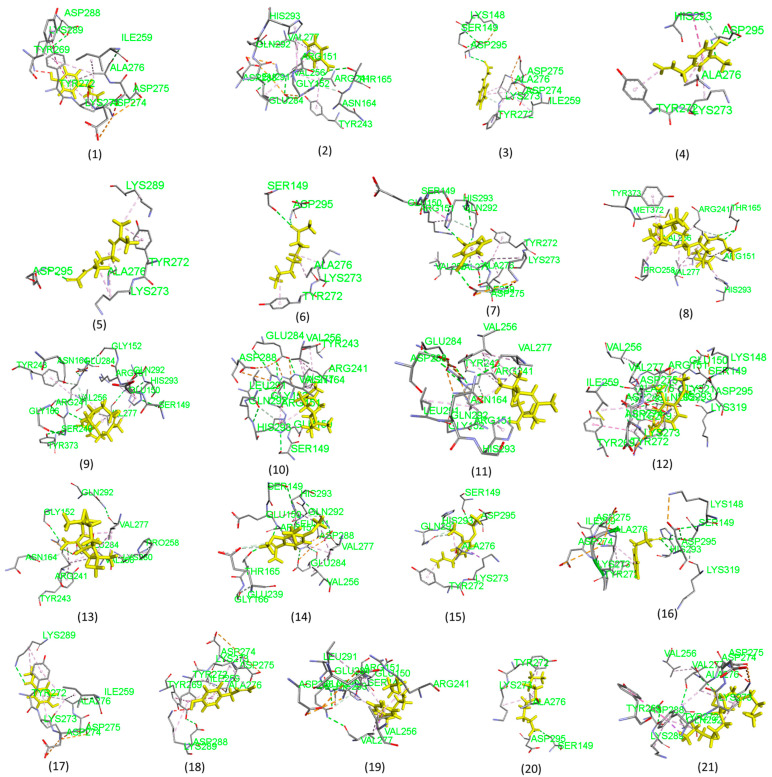
Three-dimensional plot of molecular docking results showing the interactions between the receptor (*Staphylococcus aureus*) and the ligands (compounds 1–21 from *Syzygium aromaticum*, as listed in Table 3).

**Figure 3:**
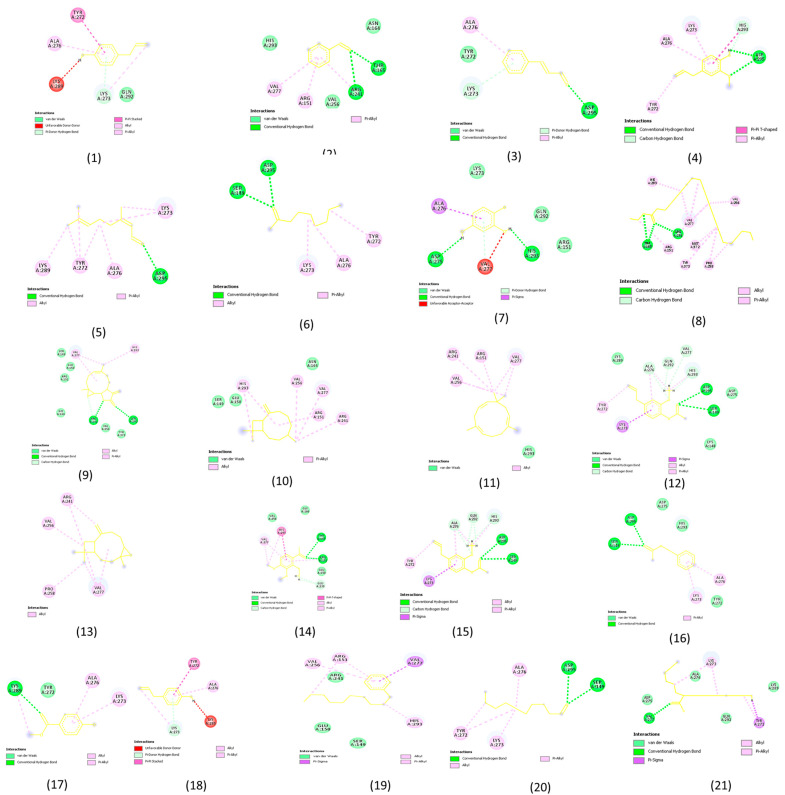
Two-dimensional plot of molecular docking results showing the interactions between the receptor (Staphylococcus aureus) and the ligands (compounds 1–21 from *Syzygium aromaticum*, as listed in Table 3).

We performed molecular docking analysis using AutoDock Vina (version 1.1. 2) (Figure 2 and Figure 3). Auto Dock Vina provides the ten best binding positions for each complex form between the receptor (*Staphylococcus aureus*) and ligands (*S. aromaticum*). We selected the best pose as indicated by the lowest docking energy score. The docking energy scores for each complex are listed in Table 3, and their corresponding protein–ligand interactions are shown in Figure 2 (3D) and Figure 3 (2D). It is noted that the compound naphtho [2,3-b] furan-2-one,3-[[2-(4-methoxy (a compound extracted from *S. aromaticum*) has the highest binding affinity of −7.2 kcal/mol. In contrast, the compound 2-nonanone (an extracted compound from *S. aromaticum*) has the lowest binding affinity of −4.1 kcal/mol. Moreover, it is observed that the binding affinity between receptors (*Staphylococcus aureus*) and ligands (*S. aromaticum*) is significant, which supports the above antibacterial experimental results.

## 3. Discussion

### 3.1. Antioxidant Properties of Clove Pods

In the ABTS radical scavenging assay, the IC_50_ value of clove extract was 0.18 ± 0.01 mg/mL, with that of BHT being 0.017 ± 3 × 10^4^ mg/mL, and that of ascorbic acid being 0.022 ± 0.001 mg. Clove extract could scavenge radicals similar to in the DPPH experiment; however, it is not as effective an antioxidant as more conventional pharmaceuticals. Clove extract’s antioxidant benefits are due, in part, to its high flavonoid concentration. However, due to its concentration or possible synergistic interactions, it may demonstrate a lower efficacy.

The goal of this experiment was to find sample concentrations that could inhibit the DPPH radical scavenging activity by half. The properties of clove extract are similar to those of butylated hydroxytoluene and ascorbic acid. Clove extract mainly contains the antioxidants eugenol and eugenyl acetate. It performed worse in multiple studies when compared to more conventional antioxidants. Clove extract, more than any other ingredient, stabilizes and enhances flavor. In tests comparing it to pure antioxidants, such as BHT and A.A., it performs worse. As a natural alternative to synthetic antioxidants, it can be used to preserve meat and cosmetics without sacrificing safety.

While clove outperforms mint leaves and sesame seeds in antioxidant tests, it lags behind walnut leaves and bilberry leaves in terms of ABTS and DPPH activity. In ABTS assays, walnut leaves had 332.36 mmol Trolox equivalents per kg, which is higher than clove’s concentration in previous studies. While clove’s flavonoid concentration is essential for its effectiveness, it is weaker than synthetic alternatives in pure testing. Cloves possess polyphenols, which play a role in their antioxidant action. The radical scavenging activity of clove extract is comparable with that of ABTS; however, it is not as powerful as that of synthetic antioxidants. Although its concentration-dependent efficacy has limitations, the IC_50_ values show that it has potential as a natural antioxidant. According to Bao et al. [22]’s findings, cloves showed great antioxidant capabilities in the in vitro tests, and also significantly reduced the generation of lipid radicals. While cloves were found to be a more effective antioxidant in roast beef, this finding suggests that cloves may have the potential to be utilized as a natural antioxidant for roasted meat products.

Because of their diverse bioactive components and higher polyphenol content, walnut and bilberry leaves outperform cloves in antioxidant testing. This is in agreement with the results, indicating that sources rich in polyphenols usually have better antioxidant activities [23].

Our results back up these claims, since the IC_50_ values for clove extract were significantly higher than those for BHT and ascorbic acid (0.017–0.024 mg/mL vs. 0.08 mg/mL for DPPH and 0.18 mg/mL for ABTS, respectively). Antioxidants derived from clove extract are effective; however, synthetic and separated antioxidants are usually more potent because they are stable and pure. In antioxidant tests, clove extract is superior to mint leaves and sesame seeds, but it is inferior to walnut and bilberry leaves. Take walnut leaves as an example—they have three hundred and thirty-two millimoles of Trolox equivalents per kilogram, which is significantly greater than that of clove pods.

Additionally, clove extract improves the color and flavor of food while stabilizing it by preventing oxidation. Clove extract might not be more effective than BHT or A.A. in pure antioxidant tests, but it has a wider variety of uses. By offering a safer and more natural alternative to synthetic antioxidants, it has further advantages in practical contexts such as meat preservation and cosmetics. Pure experiments show that synthetic antioxidants, with their more reactive chemical structures, such as ascorbic acid and beta-hydroxytryptophan, are more efficient. Gülçin et al. [24] determined that clove extract is the preferred choice for natural applications due to its safety and multifunctionality. Clove extract is a safer, more natural, and more adaptable alternative to pure synthetic antioxidants, even though it is not as powerful. Cosmetics, meat preservation, and culinary flavoring are just a few of its many common uses.

Among the bioactive compounds found in cloves is eugenol. Clove phenolics, such as eugenol, have antibacterial and antioxidant properties that are useful in food systems. For active packaging and its related systems, cloves extend the life of food while also improving its nutritional value [5].

Bioactive substances have direct effects on all forms of life. They boost nutritional value; aid the cardiovascular system, immunological system, and brain; and reduce the likelihood of developing chronic diseases. Antioxidants shield cells from damage and reduce the likelihood of developing cardiovascular and chronic diseases, as well as fighting cancer [25,26]. Clove extract’s concentration and the possibility of synergistic interactions among its bioactive components affect its effectiveness in antioxidant tests.

The phytochemical composition of clove extract often gives it antioxidant properties. Nevertheless, in trials including DPPH, ABTS, and β-carotene bleaching, it fails to surpass pure antioxidants such as BHT and A.A. Nevertheless, the food, pharmaceutical, and cosmetic industries cannot function without it, given its reputation as an all-natural antioxidant that poses fewer safety concerns. Despite being less efficient than synthetic antioxidants like BHT and ascorbic acid in controlled testing, clove extract is a powerful natural antioxidant with several uses. Because of its natural origin, versatility, and lack of side effects, it is a great substitute for artificial ingredients in cosmetics and food preservation. Its concentration and its possibility for synergistic effects on efficacy should be the subjects of future research.

Research that focuses on free radicals, plant extracts, and antioxidants produced from plants in foodstuffs and biological media has to be supported by the validation of biological markers that are intended to determine the effectiveness of antioxidant components in diets [27]. Furthermore, clove extract’s multifunctionality extends to cosmetics, where it acts as an antioxidant and antimicrobial agent, enhancing product stability and safety. While synthetic antioxidants like BHT and ascorbic acid outperform clove extract in pure assays, the latter’s broader applications and safety profile make it a compelling choice in the food and cosmetic industries [28,29].

Clove extract has distinct benefits in practical contexts, even if it does not perform as well in pure antioxidant studies. As a natural preservative, it improves sensory qualities, including color and scent, while stabilizing food by preventing lipid oxidation. The use of clove extract in meat preservation is highly advantageous because it increases the product’s shelf life and decreases the need for synthetic chemicals, which is in line with customer expectations for safer and more natural options [30,31].

Additionally, clove extract is multipurpose and can be used in cosmetics to improve the stability and safety of products by acting as an antioxidant and antibacterial agent. Although clove extract is not as effective as synthetic antioxidants, such as BHT and ascorbic acid in pure tests, it is a popular choice in the food and cosmetic industries due to its safety profile and wider range of applications [28,29].

### 3.2. GC-MS Constituents

The GC-MS analysis of clove pods demonstrated a varied phytochemical composition, with eugenol (58.86%) as the predominant compound, succeeded by caryophyllene (14.72%), phenol, 2-methoxy-4-(2-propenyl)-acetate (9.60%), humulene (3.62%), eugenyl acetate (3.13%), and n-hexadecanoic acid, methyl ester (2.05%). Eugenol’s predominance aligns with prior research that has recognized it as the principal bioactive molecule accountable for the pharmacological attributes of cloves [7]. Eugenol is esteemed for its antibacterial, anti-inflammatory, and antioxidant characteristics, rendering it a significant compound in medical, cosmetic, and food preservation contexts.

Two bioactive sesquiterpenes, caryophyllene and humulene, greatly affect the anti-inflammatory and analgesic qualities of cloves. Specifically, caryophyllene has been demonstrated to interact with CB2 receptors, which would help to explain its neuroprotective and analgesic properties [32]. These results imply the possible uses of substances derived from cloves in neuroprotection and pain relief.

Cloves have more phenolic components than cinnamon (Cinnamomum verum) and ginger (Zingiber officinale), especially eugenol, which increases their antibacterial action against bacterial and fungal infections [33]. Moreover, a major eugenol derivative, eugenyl acetate, increases the bioactivity of clove and so becomes very useful in antimicrobial uses.

The main chemical that causes many of the therapeutic, antibacterial, and fragrant effects of clove is eugenol. Other key ingredients, including caryophyllene (14.72%), eugenyl acetate (3.13%), and humulene (3.62%), also support its biological actions [34]. When taken in concert, these secondary metabolites greatly increase the therapeutic potential and synergistic bioactivity of clove.

Although the concentration of the bioactive components in clove essential oil can be affected by geographical origin, climatic conditions, and extraction methods, these well-documented advantages remain. Previous investigations show that whilst supercritical CO_2_ extraction better retains the integrity of bioactive substances [35], solvent-based extractions may cause the loss of volatile components. Thus, consistency and efficacy in clove-derived medicinal and nutraceutical products depend on perfecting the extraction techniques.

As this study reveals, clove extracts lack nitrogen- and chlorine-containing chemicals, which emphasizes their natural character. Particularly, eugenol derivative phenolic compounds and esters highlight their possible pharmacological and therapeutic uses. These results help to clarify the bioactive elements of clove, therefore supporting its usage in medicine, aromatherapy, and the culinary arts.

Likewise, our results fit, although in some ways they differ from earlier investigations. For instance, Ahamad et al. [36] reported 43 components in clove essential oil, including a comparable eugenol content (59.16%) alongside notable compounds such as β-selinene (9.34%) and α-humulene (2.16%). These variations imply that the chemical makeup of clove oil is very much influenced by the environmental conditions, plant provenance, and extraction techniques. The noted variation in caryophyllene and humulene levels between research emphasizes the importance of consistent extraction methods to guarantee consistent product compositions.

Comparative GC-MS analyses of medicinal plants reveal distinct chemical signatures that contribute to their therapeutic effects. For example, ginger (Zingiber officinale) rhizomes contain gingerol and shogaol, compounds known for their anti-inflammatory and analgesic activities. These bioactive molecules complement clove’s pharmacological profile, indicating that both plants could be utilized synergistically in traditional medicine, pharmaceuticals, and food science [37].

The well-documented antibacterial qualities of clove pods support their possible medicinal uses even further. Research by Hassan et al. [38] and Hu et al. [39] demonstrated that clove essential oil—rich in eugenol—disrupts bacterial membranes, leading to ion leakage, protein denaturation, and bacterial cell death. Clove oil has been shown to be highly effective against Staphylococcus aureus, Escherichia coli, and Salmonella typhimurium, with a minimum inhibitory concentration (MIC) as low as 0.304 mg/mL, confirming its potent antibacterial activity.

Interestingly, unlike some other natural antimicrobials, clove oil remains effective regardless of variations in bacterial membrane composition. This unique property enhances its potential use as a natural food preservative and therapeutic agent for antibiotic-resistant bacterial infections [40].

Beyond its antibacterial effects, GC-MS analysis of clove oil reveals its diverse pharmacological potential. Due to its anti-inflammatory, analgesic, and antioxidant properties, clove oil is widely used in the pharmaceutical industry, particularly in dental care products, topical analgesics, and wound healing formulations. Additionally, its bactericidal effectiveness supports its application as a natural food preservative in the food industry.

Furthermore, clove’s bioactive potential extends to agriculture, where its medicinal properties suggest applications in pest management and crop protection. By incorporating natural plant-based compounds, agricultural industries could reduce their reliance on synthetic pesticides, contributing to more eco-friendly and sustainable agricultural practices [41,42,43].

### 3.3. Molecular Docking Analysis

To better understand the molecular basis of the biological effects of bioactive chemicals, molecular docking is a computer-aided approach that may be used to forecast how these compounds will interact with specific target proteins. In order to confirm the antibacterial effects of clove (*S. aromaticum*) pods that were observed in the lab, we conducted docking analysis. To understand the molecular-level bioactivity of the clove extract, researchers can use this method to determine the binding sites and affinity of eugenol and other important clove components with bacterial components [7,42].

The main component of clove oil, eugenol, is a phenolic molecule with an excellent binding affinity for enzymes produced by bacteria, including DNA gyrase and dihydrofolate reductase. Bacterial metabolic activities and DNA replication rely on these enzymes. The antibacterial properties of clove are underscored by the fact that eugenol and its derivatives block various enzyme pathways [44]. Additionally, docking experiments have shown that clove chemicals cause paralysis and death in insect larvae by interacting with acetylcholinesterase, an essential enzyme in these organisms. This mechanism can explain the behavior of clove oil [45].

Extensive in silico and experimental research has proved clove pods’ powerful antimicrobial properties. Eugenol kills bacteria by penetrating their cell membranes and releasing vital ions and proteins. Research has shown that clove essential oil has a minimum inhibitory concentration (MIC) between 0.25 and 0.5 mg/mL against *Staphylococcus aureus*, *Escherichia coli*, and *Salmonella typhimurium* [46].

Clove oil has a broader range of antibacterial actions, particularly against foodborne pathogens, compared to other plant-derived antimicrobials like thyme or garlic. Its intense antibacterial action makes it a highly valuable natural preservative for food goods as an alternative to synthetic antimicrobials. Clove oil has also been investigated as a topical treatment for skin infections and mouth disorders, such as dental caries in clinical settings [47].

We can learn more about clove’s bioactivities when docking studies are combined with experimental validation. Clove’s antimicrobial characteristics make it a promising natural product, with applications in agriculture and pharmaceuticals, and as a possible greener substitute for synthetic chemicals. Future research could focus on investigating the potential synergistic effects of clove components with other natural products and developing formulations to maximize their performance.

Integrating docking studies with experimental validation strengthens the understanding of clove’s bioactivities [48]. Future research could explore the synergistic effects between clove compounds and other natural products, optimizing their formulations for enhanced efficacy.

## 4. Materials and Methods

### 4.1. Collection and Preparation of the Plant Extract

**Figure 4:**
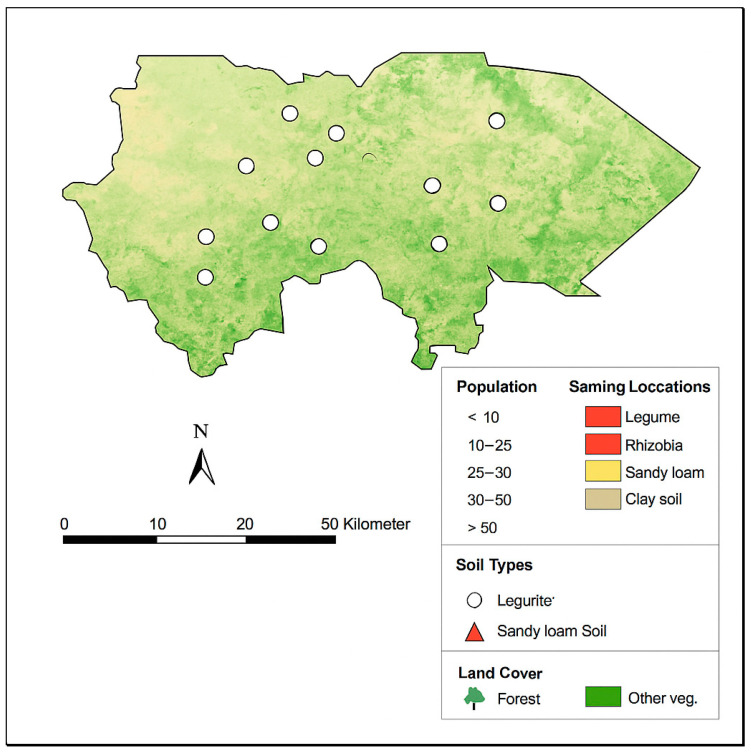
The collection site of clove pods, Wad Medani, Gezira State, Sudan.

We purchased cloves from a local market in Wad Medani, Gezira State (Figure 4), Sudan. To prepare the plant extract, we added twenty grams of the sample to one hundred and fifty milliliters of distilled water, and then we gently mixed and boiled the sample for two hours to begin the preparation process. The liquid portion of the mixture was collected by means of centrifugation at 5000 times gravity for 10 min, after passing it through a muslin cloth for filtration. The supernatant was collected, consolidated, and concentrated to one-quarter of the starting amount after two hours, and again after six hours. This systematic approach aims for the precise extraction of the target chemicals, free of degradation.

### 4.2. Antioxidant Properties

The antioxidant properties of clove pods were investigated by determining the total phenolic and total flavonoid contents, as well as through the use of a DPPH radical assay and an ABTS radical scavenging activity assay.

#### 4.2.1. Total Phenolics Measurement

The Folin–Ciocalteu phenol reagent method was modified to quantify the total phenolic content in clove pods’ methanol extracts with minor modifications [45]. In summary, 1 mL of plant extract, 1 mL of the 1N phenol reagent Folin–Ciocalteu, and 2 mL of 7% (*w*/*v*) sodium carbonate were mixed to a final volume of 10 mL using double-distilled water. A spectrophotometer was employed to determine the optical density. A standard curve was created using various gallic acid concentrations (0–100 μg/mL) and is expressed as mg GAE (gallic acid equivalents)/g fw.

#### 4.2.2. Calculating Total Flavonoids

The flavonoid concentration in the ethanol extract of clove pods was measured using the aluminum chloride reagent reported by Ordonez et al. [47]. We combined the plant extract with 1 mL of 2% ethanolic AlCl_3_ (*w*/*v*). The reaction mixture sat at 25 °C for one hour. The optical density of the golden yellow color at 420 nm was determined using a spectrophotometer (UV–Vis Spectrophotometer, Model no. 2203, Systronics, Ahmedabad, India). Quercetin (0–100 μg/mL) was utilized to create the standard curve. The plant extracts were measured for total flavonoid content using mg quercetin equivalent (Q.E.)/g fw.

#### 4.2.3. DPPH Radical Test

A total of 1 mL of the extract was mixed with 5 mL of DPPH (0.135 mM) in 80% (*v*/*v*) ethanol, and the mixture was incubated for 30 min at an ambient temperature in the dark to determine the DPPH radical scavenging activity in methanol extracts of clove bud oil [49]. The reaction mixture’s optical density was measured at 517 nm using a spectrophotometer (UV–Vis Spectrophotometer, Model 2203, Systronics, India). One milliliter of methanol and five milliliters of DPPH were used as a blank. We assessed the DPPH inhibition potential of several extracts as percentages (%) using the following equation:

DPPH Inhibition % = (A_b_ − A_s_/Ab) × 100

where

A_b_ = the absorbance of the blank;

A_s_ = the absorbance of the sample.

#### 4.2.4. ABTS Radical Scavenging Activity Assay

The 2,2′-casino-bis (3-ethylbenzthiazoline-6-sulphonic acid) cation scavenging activity test was used for the antiradical assay [49]. Reacting 7 mM ABTS solution with 2.45 mM K_2_S_2_O_8_ produced radical monocation. The combination sat for 15 h at room temperature in the dark. Organic and aqueous extracts were dissolved in methanol and distilled water, respectively.

Different extract and tocopherol (vitamin E) concentrations were investigated. The standard was used for comparison. The antioxidant activity was measured by adding 200 µL of each standard and sample to 800 µL of diluted ABTS·+.

After 30 min, the absorption was measured at 734 nm using spectrophotometry. Triplicate measurements were taken. The antioxidant capacity of the test samples and the standard is presented as % inhibition. The ABTS^+^ scavenging activity (%) was calculated using the following equation:

PI% = 100 × (AControl − ASample)/AControl,

where AControl and ASample represent the absorbances of the control and the test sample/standard, respectively.

### 4.3. β-Carotene/Linoleic Acid Method

The β-carotene bleaching inhibition of the extracts was measured using a method reported by Ikram et al. [50]. Heating a β-carotene/linoleic acid combination results in the production of a free radical. A 2 mL volume of β-carotene solution (1.5 mg/2.5 mL chloroform) was mixed with 20 μL of linoleic acid and 200 μL of tween-20. The chloroform was vacuum removed at 40 °C using a rotary evaporator. A 50 mL volume of distilled water was added to the dried mixture to create a β-carotene/linoleic acid emulsion. The β-carotene bleaching activity of each extract was measured by adding 0.800 mL of emulsion to 0.200 mL of extracts at various concentrations (20 mg/mL) and the standard (1 mg/mL). The mixes were incubated at 50 °C for 120 min and measured at 470 nm before and after incubation. Triplicate tests were carried out. The antioxidant activity of the extract was estimated using this equation:

PI% = [1 − (A0 − At/Ac0 − Act)] × 100,

where A0 and Ac0 are the absorbance values at zero time for the test sample or the standard and the control, respectively, and At and Act are the results after 120 min of incubation.

### 4.4. GC-MS Analysis

The bioactive compounds of the clove bud extract were assessed using a gas chromatography–mass spectrometer, as described by Aggarwal et al. [51].

The DB-5 column was thirty meters long, with an inside diameter of twenty-five millimeters and a thickness of twenty-five micrometers. Helium, working at a flow rate of 1.1 mL/min, was utilized as the carrier gas. The injection temperature and detection were set at 250 °C, and a temperature program that ranged from 40 to 460 °C was implemented, with an increasing trend of 5 °C per minute. A 0.2 mL injection volume and an electronic ionization detector with an ionization energy of 70 electron volts were utilized in this experiment.

### 4.5. Molecular Docking

#### 4.5.1. Protein Structure Preparation

We retrieved the crystal structure of *Staphylococcus aureus* protein (PDB ID: 5M18) from the protein databank to depict the antimicrobial activity of *S. aromaticum*. Moreover, the crystal structure of *Ades Agepty protein* (PDB ID: 7EBT) from the protein databank was used to confirm the larvicidal activity of *S. aromaticum*. Further, the above pdb structures were separated from complex 3D structures using Discovery Studio Version 2020.

The structures of various clove pod compounds, such as phenol,4-(2-propenyl)-, benzaldehyde, 2-propenal, 3-phenyl-, eugenol, bicycle [3,1-1]heptan-3-ol, 2-nonanone, 1,2,3-benzeneetriol, docosanoic acid, ethyl ester, naphtho [2,3-b]furan-2-one,3-[[2-(4-methoxy, caryophyllene, humulene, phenol, 2-methoxy-4-(2-propenyl)- acetate, caryophyllene oxide, 2,3,4-trimethoxyacetophenone, eugenyl acetate, acetic acid, phenylmethyl ester, methyl salicylate, chavicol, 1-propyl-3(popen-1-yl)adamantine, decanal, and n-hexadecanoic acid, and methyl ester were drawn with the help of ChemBioDraw ultra 14 software.

#### 4.5.2. Performing Molecular Docking

We performed molecular docking using AutoDock Vina tools to determine the receptor–ligand interactions. For the ligand binding site of NQO1, we fixed the grid box parameters with X = 52, Y = 56, and Z = 80 (center grid box: X = 2.384, Y = −1.009, Z = 3.269; spacing = 0.347Angstrom) dimensions. Moreover, we used the AutoDock Vina tool to carry out all the docking procedures with the predetermined parameters mentioned above. Further, we visualized the receptor–ligand interaction using the Discovery Studio 4.0 client.

### 4.6. Statistical Analysis

Descriptive statistical approaches were applied in data analysis. Treatments’ notable variations were found using the least significant difference (LSD) test. Plotting the mortality rate against the dosage of a substance, this approach uses probit units to modify the mortality data and subsequently finds the dosages (LD50 and LD95) that match the 50% and 95% death rates. This helps to assess the toxicity of the substance under study.

## 5. Conclusions

Finally, clove pods’ phytochemical and antioxidant characteristics, as well as GC-MS studies on their properties, suggest enormous potential. This research reveals the enormous potential of plants to offer new therapeutic and environmental solutions. We must promote further research into the benefits intrinsic to nature. By using the rich knowledge attainable through botany, we can improve health, ecology, and sustainability.

Our efforts to promote natural treatments and plant-based compounds will lead to remarkable discoveries and revolutionary developments driven by the relentless quest for medical advances and ecological sustainability. Clove pods show promise in various fields, but many challenges and research opportunities remain. Researchers struggle to replicate clove plant growth. It is necessary to appropriately analyze the effects of the plant’s phytochemicals on human health and disease. In vitro and in silico clove compound–human interaction models must be improved to circumvent dangerous and time-consuming investigations. Clove derivatives may cure fungal infections, neurological diseases, and cancer. Regarding practical implications, clove extract, though less effective than typical antioxidants, offers a natural and safer option for antioxidant-based applications like food preservation and nutraceuticals.

## Figures and Tables

**Table 1:** Antioxidant activities of clove extract compared to known drugs.

Test System	Extract	Butylated Hydroxytoluene	Ascorbic Acid
Phytochemical screening			
1. Total Phenols (mg GAE/g Extract)	7.25 ± 0.12	-	-
2. Total Flavonoids (mg QE/g Extract)	57.22 ± 0.41	-	-
Antioxidant Assays			
1. DPPH IC_50_ (mg/mL)	0.08 ± 0.01	0.024 ± 2 × 10^−4^	0.021 ± 5 × 10^−4^
2. ABTS IC_50_ (mg/mL)	0.18 ± 0.01	0.017 ± 3 × 10^−4^	0.022 ± 0.001
3. β-carotene IC_50_ (mg/mL)	1.78 ± 0.11	0.044 ± 3.2 × 10^−3^	0.019 ± 0.001

**Table 2:** GC-MS of clove (*S. aromaticum*) pods.

Peak	R. Time	Area %	Compound Name	Mol. Form.
1	7.564	0.39	Phenol,4-(2-propenyl)-	C_9_H_10_O
2	7.810	0.53	Benzaldehyde	C_7_H_6_O
3	8.029	0.31	2-propenal, 3-phenyl-	C_9_H_8_O
4	8.231	41.4	Eugenol	C_10_H_12_O_2_
5	8.633	1.13	Bicycle [3,1-1]heptan-3-ol	C_10_H_16_O
6	9.120	0.32	2-nonanone	C_9_H_18_O
7	9.166	0.68	1,2,3-benzeneetriol	C_6_H_6_O
8	9.566	0.11	Docosanoic acid, ethyl ester	C_24_H_34_O_2_
9	9.599	0.14	Naphtho [2,3-c]furan-1,3-dione	C_12_H_6_O_3_
10	10.050	10.42	Caryophyllene	C_15_H_24_
11	10.385	2.73	Humulene	C_15_H_24_
12	10.702	7.21	Phenol, 2-methoxy-4-(2-propenyl)-acetate	C_12_H_14_O_3_
13	11.584	0.78	Caryophyllene oxide	C_15_H_24_O
14	12.337	0.69	2,3,4-trimethoxyacetophenone	C_11_H_14_O_4_
15	12.541	2.22	Eugenyl acetate	C_12_H_14_O_3_
16	13.045	0.54	Acetic acid, phenylmethyl ester	C_9_H_10_O_2_
17	13.641	0.17	Methy salicylate	C_8_H_8_O_3_
18	14.326	0.36	Chavicol	C_10_H_18_O
19	14.675	1.54	1-propyl-3(popen-1-yl)adamantine	C_16_H_26_
20	16.283	0.76	Decanal	C_10_H_20_O
21	17.023	2.14	n-hexadecanoic acid, methyl ester	C_16_H_32_O_2_
Total		100		

**Table 3:** Binding affinities of the top-rated docking pose for receptor (Staphylococcus aureus) and ligand (*Syzygium aromaticum)* complexes, measured in kcal/mol.

SN. NO.	Receptor	Compound Name	Binding Energy (kcal/mol)
1	*Staphylococcus aureus* (PDB ID: 5M18)	Phenol,4-(2-propenyl)-	−5.1
2		Benzaldehyde	−4.7
3		2-propenal, 3-phenyl-	−5.2
4		Eugenol	−5.4
5		Bicycle [3,1-1]heptan-3-ol	−5.6
6		2-nonanone	−4.7
7		1,2,3-benzeneetriol	−5.0
8		Docosanoic acid, ethyl ester	−4.6
9		Naphtho [2,3-b]furan-2-one,3-[[2-(4-methoxy	−7.2
10		Caryophyllene	−6.8
11		Humulene	−6.5
12		Phenol, 2-methoxy-4-(2-propenyl)- acetate	−5.6
13		Caryophyllene oxide	−6.8
14		2,3,4-trimethoxyacetophenone	−5.2
15		Eugenyl acetate	−6.0
16		Acetic acid, phenylmethyl ester	−5.4
17		Methy salicylate	−5.3
18		Chavicol	−5.1
19		1-propyl-3(popen-1-yl)adamantine	−5.0
20		Decanal	−4.9
21		n-hexadecanoic acid, methyl ester	−5.5

## Data Availability

All data generated or analyzed during this study are included in this published article.
